# Contributions of the Catechol-O-Methyltransferase Val158Met Polymorphism to Changes in Brain Iron Across Adulthood and Their Relationships to Working Memory

**DOI:** 10.3389/fnhum.2022.838228

**Published:** 2022-04-27

**Authors:** Jonatan Gustavsson, Goran Papenberg, Farshad Falahati, Erika J. Laukka, Grégoria Kalpouzos

**Affiliations:** ^1^Aging Research Center, Karolinska Institutet and Stockholm University, Stockholm, Sweden; ^2^Stockholm Gerontology Research Center, Stockholm, Sweden

**Keywords:** brain iron, COMT, working memory, longitudinal, ageing, QSM, dopamine, SEM

## Abstract

Ageing is associated with excessive free brain iron, which may induce oxidative stress and neuroinflammation, likely causing cognitive deficits. Lack of dopamine may be a factor behind the increase of iron with advancing age, as it has an important role in cellular iron homoeostasis. We investigated the effect of *COMT* Val 158 Met (rs4680), a polymorphism crucial for dopamine degradation and proxy for endogenous dopamine, on iron accumulation and working memory in a longitudinal lifespan sample (*n* = 208, age 20–79 at baseline, mean follow-up time = 2.75 years) using structural equation modelling. Approximation of iron content was assessed using quantitative susceptibility mapping in striatum and dorsolateral prefrontal cortex (DLPFC). Iron accumulated in both striatum and DLPFC during the follow-up period. Greater iron accumulation in DLPFC was associated with more deleterious change in working memory. Older (age 50–79) Val homozygotes (with presumably lower endogenous dopamine) accumulated more iron than older Met carriers in both striatum and DLPFC, no such differences were observed among younger adults (age 20–49). In conclusion, individual differences in genetic predisposition related to low dopamine levels increase iron accumulation, which in turn may trigger deleterious change in working memory. Future studies are needed to better understand how dopamine may modulate iron accumulation across the human lifespan.

## Introduction

Ageing is associated with decline in cognition that differs in degree and pace between individuals (Kirkwood et al., 2005; Rönnlund et al., 2005; De Frias et al., 2007; Raz and Kennedy, 2009; Nyberg et al., 2012). Ageing is also accompanied by neural alterations at the structural, functional and molecular levels, including iron accumulation (Raz et al., 2003, 2005; Bartzokis, 2004; Fjell and Walhovd, 2010; Grady, 2012; Daugherty et al., 2015; Kalpouzos et al., 2017; Nyberg et al., 2020).

Intracellular non-heme iron is important for fundamental neurobiological processes such as myelination (Connor and Menzies, 1996; Todorich et al., 2009), synthesis of adenosine triphosphate (ATP) in mitochondria (Mills et al., 2010), and dopamine metabolism (Mills et al., 2010; Hare and Double, 2016). Iron is bound to ferritin and released when needed. During ageing, free iron accumulates outside the binding complexes and excessive amount of free iron induces oxidative stress *via* Fenton’s reaction (Winterbourn, 1995). This is particularly detrimental to cells as iron-induced oxidative stress disrupts mitochondrial function, which in turn, can lead to further release of iron from ferritin in an attempt to return to normal function and iron homoeostasis (Ward et al., 2014). A positive-feedback loop of iron-induced oxidative stress is formed and exacerbates the toxic effects of iron that ultimately leads to cell apoptosis *via* inflammation (Zecca et al., 2004; Mills et al., 2010; Williams et al., 2012; Ward et al., 2014). Thus, the deleterious cascade of iron-related oxidative stress highly depends on accumulation of free iron in the cells.

In older age, excessive iron load has deleterious consequences for brain structure and function (Daugherty et al., 2015; Daugherty and Raz, 2016). More specifically, greater iron content has previously been linked to striatal atrophy (Daugherty et al., 2015; Daugherty and Raz, 2016), poorer myelin integrity (Steiger et al., 2016), reduced task-related frontostriatal activity related to memory (Kalpouzos et al., 2017; Salami et al., 2021), and reduced striatal resting-state connectivity (Salami et al., 2018). Moreover, higher brain iron load has been associated with reduced cognitive abilities (Daugherty and Raz, 2015; Kalpouzos, 2018), including memory (Bartzokis et al., 2011; Rodrigue et al., 2013; Kalpouzos et al., 2017), executive functions (Ghadery et al., 2015), and psychomotor performance (Adamo et al., 2014; Ghadery et al., 2015; Salami et al., 2018). Longitudinal studies on the iron-cognition link are scarce. More specifically, greater baseline iron predicted less working memory improvement (Daugherty et al., 2015), and increased iron concentration with worse executive function performance at follow-up (Blasco et al., 2017).

Age-related decline in the dopamine system (Bäckman et al., 2006) may be a factor behind the increase of free iron with advancing age. Iron is essential for dopamine synthesis as a co-factor to tyrosine hydroxylase in substantia nigra and ventral tegmental area (Zucca et al., 2017) and co-localised with dopamine vesicles (Ortega et al., 2007). On the other hand, dopamine has an important role in cellular iron homoeostasis. *In vitro* studies showed that dopamine may be protective against iron-induced oxidative stress by increasing the uptake of free iron (Dichtl et al., 2018). Furthermore, dopamine receptor activation may alleviate and suppress neuroinflammation (Shao et al., 2013; Yan et al., 2015; Zhu et al., 2018; Liu et al., 2021). At the same time, previous research has shown dopamine to cause the release of ferritin-bound iron (Double et al., 1998; Tanaka et al., 1999). At low concentrations of dopamine, the released iron can induce oxidative damage to the cell. However, at high concentrations of dopamine, this oxidative damage is inhibited (Tanaka et al., 1999).

Motivated by the lack of human studies investigating the link between dopamine and brain iron, as well as scarcity of longitudinal studies on brain iron accumulation, we aimed to investigate the relationships among dopamine, iron, and working memory in relation to age. First, we hypothesised that greater iron at baseline and accumulation of iron would be linked to higher working memory decline. Second, we hypothesised that lower levels of dopamine should be associated with greater brain iron accumulation and working memory decline, exacerbated by age-related dopamine decline. We used data from a longitudinal adult lifespan sample (IronAge, *n* = 208, age = 20–79) to investigate whether individuals carrying genetic predisposition related to low dopamine levels accumulate more iron, which in turn may lead to higher working memory decline. More specifically, we focussed on a well-investigated genetic variation in the Catechol-O-methyltransferase (*COMT* rs4680) gene, crucially implicated in central dopamine function (Witte and Flöel, 2012). The COMT enzyme is involved in extracellular degradation of synaptically released dopamine in the frontal cortex (Matsumoto et al., 2003; Chen et al., 2004), but also in striatum (Matsumoto et al., 2003). The activity of the COMT enzyme modulates dopamine concentration, which in turn affects the neural signal-to-noise ratio crucial for efficient cognitive processing (Egan et al., 2001). The *COMT* Val/Val genotype has been related to lower dopamine levels due to higher turnover activity (Lotta et al., 1995; Chen et al., 2004), resulting in worse executive functioning (De Frias et al., 2005) and working memory performance (Papenberg et al., 2014) compared to Met carriers, who in turn demonstrated higher accuracy (Störmer et al., 2012) and faster response times (Nagel et al., 2008) during working memory performance. To approximate iron content, we used the recently developed technique quantitative susceptibility mapping (QSM) for a quantitative estimate of tissue magnetic susceptibility.

## Materials and Methods

This study uses data from the IronAge project, which was approved by the Regional Ethical Review Board in Stockholm. All participants provided written informed consent prior to data collection.

### Participants

The participants were recruited through advertisements in newspapers and dedicated student websites around Stockholm metropolitan area. Two hundred and thirty-two individuals were recruited for baseline data collection. All participants were fluent in Swedish, right-handed, and reported no history of neurological or psychiatric conditions. Nine individuals were excluded due to incidental brain abnormalities and fifteen were excluded due to dropping out of the protocol. This resulted in a baseline sample of *n* = 208 (108 female). After approximately 3 years (Mean days between scans: 1,003, SD: 81), 151 individuals from the baseline sample expressed interest in undertaking the follow-up protocol. Of those, 135 were eligible to participate (i.e., they had no new health conditions incompatible with the study) and completed the full protocol. We examined the influence of selective attrition by comparing returning participants and drop-outs on demographic variables and working memory performance. The comparison revealed only a significant difference with respect to age (*t* = −2.06, *p* = 0.041) with drop-outs being younger than returning participants. Table 1 shows participants’ characteristics.

**TABLE 1:** Participant characteristics for the total sample and the sample with available follow-up data.

		Baseline *n* = 208		Baseline *n* = 135		Follow-up *n* = 135	
		Mean	SD	Mean	SD	Mean	SD
Age		50.00	17.28	52.00	16.33	54.67	16.30
MMSE		28.42	1.35	28.45	1.38	28.84	1.39
Volume^a^							
Striatum		17,316	1,588	17,343	1,579	17,228	1,626
DLPFC		45,804	4,856	45,618	4,941	45,431	4,795
Iron^b^							
Striatum		0.102	0.026	0.104	0.025	0.111	0.023
DLPFC		0.041	0.012	0.042	0.012	0.045	0.012
*WM*		13.56	1.46	13.62	1.29	14.14	1.33
Younger		14.40	1.13	14.36	1.16	15.06	1.12
Age range	20–49						
Older		12.80	1.31	13.04	1.09	13.42	1.01
Age range	50–79						
*COMT*							
Met/Met		54		35			
Met/Val		92		60			
Val/Val		53		33			

*MMSE, Mini Mental State Examination; DLPFC, Dorsolateral prefrontal cortex; WM, Working memory; SD, Standard deviation.*

*^a^Volume (mL) adjusted for total intracranial volume.*

*^b^Approximation of iron, based on susceptibility in parts per million.*

### Genotyping

DNA was extracted from peripheral blood samples and stored at Karolinska Institutet Biobank. DNA samples were transferred on PCR plates and sent to the SNP&SEQ Technology Platform, Uppsala University [National Genomics Infrastructure (NGI), SciLifeLab Sweden]. The genotyping was performed using a multiplexed primer extension (SBE) chemistry of the iPLEX assay with detection of the incorporated allele by mass spectrometry with a MassARRAY analyser from Agena Bioscience (Ross et al., 1998; Gabriel et al., 2009; Storm et al., 2019). Raw data from the mass reader was converted to genotype data using the Typer software (Agena Bioscience). The *COMT* genotype distributions were in Hardy-Weinberg equilibrium (*p*s > 0.1). Of the full sample, 9 were missing genotype information and were treated as missing at random. Genotype distributions are reported in Table 1.

### MRI Acquisition and Preprocessing

#### Acquisition

Participants were scanned on a GE Discovery MR750 3.0T scanner, equipped with an 8-channel phased array receiving coil, at the MR center, Karolinska University Hospital, Stockholm. A structural T1-weighted 3D IR-SPGR image was obtained with the following parameters: repetition time (TR) = 6.96 ms, echo time (TE) = 2.62 ms, 176 axial slices with slice thickness of 1 mm, in-plane resolution = 0.94 × 0.94 mm^2^, field of view (FOV) = 24 cm, flip angle = 12°. For brain iron quantification, a 3D multi-echo Gradient Recalled Echo (meGRE) sequence was used with the following parameters: TR = 27.2 ms, 124 axial slices of 1.2 mm thickness, in-plane resolution = 0.94 × 0.94 mm^2^, FOV = 24 cm, flip angle = 17°. The first TE was 1.9 ms, and it was followed by 7 consecutive TEs with a constant interval of 3.18 ms between them.

#### Quantitative Susceptibility Mapping

**FIGURE 1:**
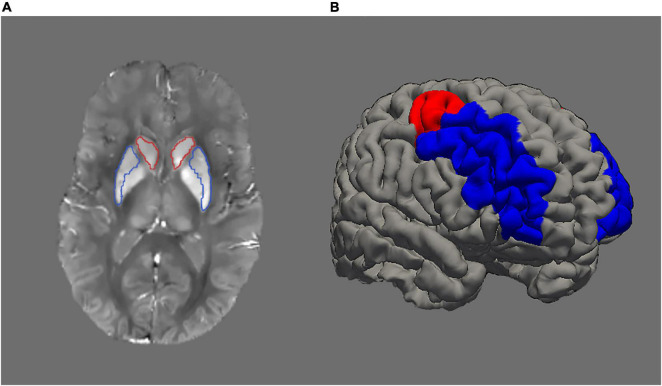
**(A)** Example of a QSM (quantitative susceptibility mapping) image. Higher signal intensity denotes higher iron load. Striatum is represented by caudate (red outline) and putamen (blue outline). **(B)** Image illustrating dorsolateral prefrontal cortex with rostral middle-frontal cortex in blue colour and caudal middle-frontal cortex in red colour.

Approximation of iron content was inferred from susceptibility values derived from QSM images. Morphology-enabled dipole inversion (MEDI; Liu et al., 2011) is a method for QSM estimation that selects the solution that minimises the discrepancy in the number of voxels belonging to edges between the susceptibility image and the magnitude image. Here, we used the recommended non-linear variant of MEDI as proposed by Liu et al. (2013). Initially, the total field map was estimated from the complex meGRE images by performing a non-linear least-square fitting on a voxel-by-voxel basis. The resulting frequency map was then spatially unwrapped using a guided region-growing unwrapping algorithm (Xu and Cumming, 1999). The background fields, that is, the superimposed field contributions that are not caused by the sources inside the brain and mainly generated by air-tissue interferences, were eliminated using a non-parametric technique based on Projection onto Dipole Fields (PDF: Liu et al., 2011). Finally, the corrected frequency map was used as input for the field-to-source inverse problem to calculate the map of susceptibility. The MEDI Toolbox^1^ was used to calculate QSM images (Figure 1A).

Due to the singularity of dipole kernel at the centre of k-space, the generated QSM images contain relative susceptibility values. Therefore, the QSM images may not necessarily be comparable across subjects in a cohort. A typical approach to address this issue is zero-referencing where a common tissue is chosen as a reference and its average susceptibility is subtracted from the susceptibility values of other tissues. In this study, a region in corticospinal tract [MNI coordinates (-24; -27; 39)] was selected as the reference region (Garzón et al., 2017). The corticospinal tract is a reliable reference point as it is resilient to age-related degeneration (de Groot et al., 2015) and shows stable susceptibility across age in adulthood (Li et al., 2014). Using white-matter areas as reference regions has previously been recommended due to their low standard deviations of susceptibility, indicating low inter-subject variation similar (Straub et al., 2017) or even lower than CSF (Deistung et al., 2013). To perform zero-referencing on each participant, the selected coordinate in MNI space was mapped first to individual space using a non-linear registration warp. Then, using an in-house developed region-growing algorithm, a region of 1,000 voxels centred on the mapped coordinate was created. The algorithm uses the white-matter mask to ensure the created region encompassed white-matter tissue only. The FMRIB Software Library (FSL)^2^ was used to calculate non-linear transformation parameters (Andersson et al., 2007) and to obtain the white-matter mask (Zhang et al., 2001).

The longitudinal stream (Reuter et al., 2012) in Freesurfer image analysis suite—version 7.1.0^3^ was used for the automated segmentation of cortical and deep grey-matter structures using T1-weighted images. The longitudinal processing pipeline creates an unbiased within-subject template space and image (Reuter et al., 2010; Reuter and Fischl, 2011) and uses the unbiased template information as an initial guess for the segmentation and surface reconstruction, which improves the robustness and reliability of the overall longitudinal analysis (Reuter et al., 2012). For each subject with two time-point MRI data, the unbiased within-subject template was created using the baseline and follow-up scans. Next, the template was used to initiate surface reconstruction and segmentation. Moreover, we included subjects with a single time-point MRI data to leverage the valuable information of the whole cohort. To this end, single time-point data went through the same longitudinal processing steps, i.e., the unbiased template was created by passing the baseline scan to the template creation step and the template used to initiate surface reconstruction and segmentation.

Next, QSM and the segmentation results were resampled to the native structural space. Then, statistics including average and standard deviation were computed on the QSM maps. We merged the segmented caudate and putamen regions from the left and right hemispheres to form striatum. Similarly, segmented rostral and caudal middle frontal regions from left and right hemispheres were merged to form dorsolateral prefrontal cortex (DLPFC; Figure 1B).

Prior to computing statistics on the QSM maps, the boundary of segmentations was eroded by one voxel, and a fraction (15%) of the most extreme values was removed to avoid the influence of high signal from neighbouring vessels and obtain more robust estimates (Garzón et al., 2017).

Relaxometry was also available in the study (see Salami et al., 2021). However, R2* has been observed to have poor reliability in regions outside the basal ganglia (Wenger et al., 2021). For comparative purpose, *post hoc* analyses showed a good intraclass correlation (ICC; Koo and Li, 2016 for interpretation of ICC) of 0.798 for QSM in DLPFC, which was greater than R2* (0.730). As our analyses included not only the striatum but also DLPFC, we therefore favoured the use of QSM over R2*.

### Working Memory

Working memory performance was assessed with a feature-binding task and the numerical N-back task administered on a computer using E-Prime 2.0 software (Psychology Software Tools, Pittsburgh, PA).

The binding task assessed the ability to associate visuospatial features in working memory (Lecouvey et al., 2015). Participants had to match 5 coloured uppercase letters with the location of 5 crosses of the same colour in a 4 × 5 grid that was displayed for 5 s. After a 8-s delay, the participants had to determine whether a black lowercase letter was presented in the correct location corresponding the cross matching the same colour. The task contained 20 trials, and performance was number of correct answers.

In the numerical n-back task, a sequence of single numbers appeared on the screen. During every item presentation, subjects indicated whether the digit on the screen was the same as the one shown 1, 2, or 3 digits back. Three blocks for each condition (1-, 2-, 3-back) were performed. Performance was calculated as proportion of false alarms subtracted from proportion of hits for 2- and 3-back only (for details about the tasks, see Kalpouzos et al., 2021).

### Statistical Analyses

To address our research questions, we used structural equation modelling (SEM) in AMOS (IBM SPSS 26). Longitudinal changes for brain iron and working memory performance were modelled by estimating change regression models (McArdle, 2009). Note that the change regression model accommodates the estimation of difference scores independent of the individuals’ initial baseline levels. The model allows to attenuate potential effects pertaining to regression to the mean, a statistical phenomenon, which may be misinterpreted as true change. Regression to the mean may be reflected in strong correlations between baseline and change (Barnett et al., 2005).

**FIGURE 2:**
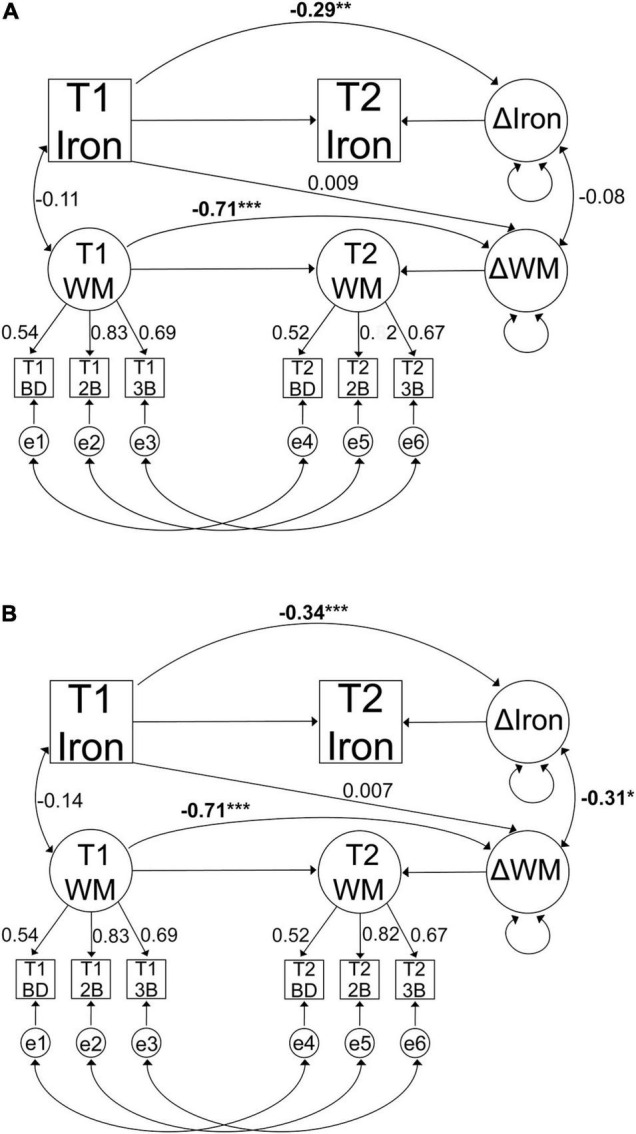
Graphical representation of the change regression model for iron accumulation and working memory change in **(A)** striatum and **(B)** DLPFC. Observed variables are indicated by boxes and latent factors are indicated by circles. Regressions are indicated with one-headed arrows and covariances with two-headed arrows. Figures present standardised parameter estimates. All models were adjusted for age, sex, and education, as well as change in grey-matter volume in case of iron accumulation. T1, baseline; T2, follow-up; e, error term; WM, working memory; BD, binding task; 2B, 2-back task; 3B, 3-back task. (*p < 0.05, **p < 0.01, ***p < 0.001).

First, we tested whether iron accumulated in striatum and DLPFC over time in separate models. Second, three indicators of working memory were used to form a latent change regression model to assess change in cognitive performance. Third, to investigate the iron-cognition link and to test our hypothesis of the effect of *COMT* on baseline iron and working memory, iron accumulation and working-memory change, we combined the first two models into one model. Figures 2A,B illustrate this measurement model for both regions. *COMT* genotype status was included as two separate effect coded predictors for baseline iron and working-memory performance, as well as for the difference scores between baseline and follow-up for iron and working memory. The *COMT* predictors were set up to contrast Met homozygotes against heterozygotes (dummy 1), and Met homozygotes against Val homozygotes (dummy 2). In addition, *COMT* genotype status and its interaction with age were included as predictors for iron and working memory difference scores. Lastly, baseline iron was also included as a predictor for working-memory change.

As mentioned above, cognitive performance at baseline and follow-up, and the difference between these two time points, were estimated in latent space. Variance common to the indicators (i.e., performance measures at baseline and follow-up) is represented as latent information whereas remaining variance is considered error variance. In these models, the residual variances of the same indicator are allowed to covary over time. Crucially, the mean and the variance of the differences between the two time points, as well as the covariance of the baseline scores with the changes were estimated from the observed indicators by including them in the model as parameters. To accommodate for missing data (e.g., due to the attrition between time 1 and 2), full information maximum likelihood (FIML; Finkbeiner, 1979; Schafer and Graham, 2002) estimates were used for all models. FIML is a procedure which uses available information for estimating parameters that contain variables with missing values rather than imputing or omitting data. Using FIML allows for a more accurate population estimate and a better measurement model for working memory compared to other procedures dealing with missing data (Schafer and Graham, 2002). The estimates are unbiased under the assumption of missing at random. That is, the likelihood of data from a variable missing may depend on other variables in the model rather than the variable itself (Little and Rubin, 2002). In our sample, only age was a significantly differentiating variable between drop-outs and returnees, whereas other important variables such as iron content and memory performance were not. We presumed the assumption of missing at randomness was met by including chronological age, but also other powerful predictors of missingness, such as sex, and education in our models to account for the largest part of the missing data (Rubin, 1975; Schafer and Graham, 2002). Note that SEM analyses restricted to returning participants only showed similar patterns as using full sample (data not reported).

More specifically, age, sex, and education were included in the models as predictors of baseline iron and working memory performance, as well as the difference scores. Furthermore, regional volumetric changes were included in the models as predictors of change in iron, as volume may be related to both age and iron content. The variables age, education, and changes in volume were centred around the mean to prevent multicollinearity and allow easier interpretation of parameter estimates.

The following indices were used to evaluate whether the model provided a good representation of the data: the comparative fit index (CFI) and the root mean square error of approximation (RMSEA). Values greater or equal 0.95 of CFI and lesser or equal to 0.08 for RMSEA were considered to indicate good fit (Kline, 2005). To accurately interpret the latent difference score as the difference in a latent construct that was measured similarly at both time points, measurement invariance was established. We first constrained the loadings of the latent factor to be equal over time-points, followed by constrained intercepts, and residual variance, assuming metric, strong, and strict invariance, respectively. We followed the recommendations made by Chen (2007) regarding model fit. A difference in model fit for the more constrained model expressed as ≥ −0.010 in CFI and ≥ 0.015 in RMSEA would indicate lack of invariance.

Both univariate (±3.29 SD; *n* = 3) and multivariate outliers (Mahalanobis’ distance; *p* < 0.001 threshold for the χ^2^ value; *n* = 5) were excluded from analyses and treated as missing (Tabachnick and Fidell, 2013). The alpha level for statistical decisions regarding significance of χ^2^ fit statistics was set to 0.05.

## Results

### Iron Accumulation in Striatum and Dorsolateral Prefrontal Cortex

**FIGURE 3:**
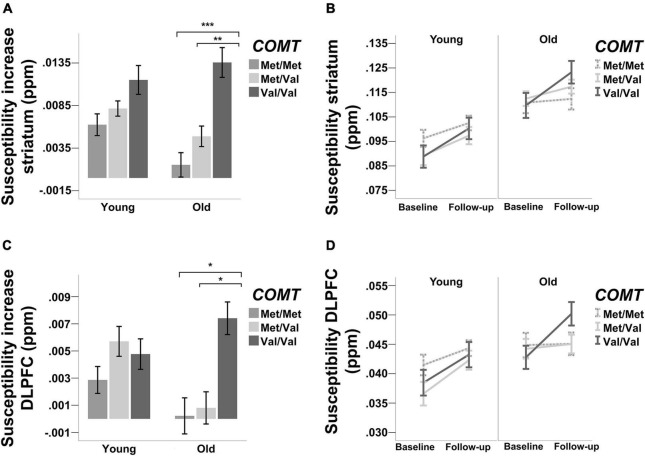
Accumulation of iron [susceptibility increase in parts per million (ppm)] in **(A,B)** striatum and **(C,D)** DLPFC. Error bars ± 1 SE. * indicates significant difference in iron accumulation based on structural equation modeling (*p < 0.05, **p < 0.01, ***p < 0.001).

Changes in regional iron were estimated in change regression models. The model for striatum exhibited an excellent fit [χ^2^ (5, *n* = 208) = 4.6, *p* = 0.47, CFI = 1.00, RMSEA = 0.00 90% CI: 0.00–0.09]. There was a significant increase in iron over time [Mean increase (susceptibility in ppm) = 0.018, *p* < 0.001] and variance in change was significant (*p* < 0.001), indicating interindividual differences in change. Figures 3A,B illustrate iron accumulation in striatum. Furthermore, baseline iron was negatively associated with iron accumulation (β = −0.292, *p* = 0.003), suggesting that more baseline iron was related to less increase. As expected, age was positively correlated with baseline iron (β = 0.573, *p* < 0.001). In contrast, age was not correlated with changes in iron (β = −0.029, *p* = 0.769).

The model for DLPFC exhibited an excellent fit as well [χ^2^ (5, *n* = 208) = 0.62, *p* = 0.99, CFI = 1, RMSEA = 0 90% CI: 0.00–0.00]. Iron increase was significant over time [Mean increase (susceptibility in ppm) = 0.013, *p* < 0.001], with significant variance in change (*p* < 0.001). Figures 3C,D illustrate iron accumulation in DLPFC. Baseline iron was negatively associated with iron accumulation (β = −0.337, *p* < 0.001), but positively associated with age (β = 0.346, *p* < 0.001). Similarly to the striatum, age was not significantly associated with changes in iron (β = −0.047, *p* = 0.575).

### Working Memory Change Over Time

The model for working memory change exhibited an excellent fit, establishing strict invariance for working memory over time [χ^2^ (27, *n* = 208) = 17.57, *p* = 0.92, CFI = 1, RMSEA = 0.00 90% CI: 0.00–0.020]. The factor loadings values for working memory indicators are reported in Figures 2A,B. Working memory performance improved over time (Mean increase = 6.282, *p* < 0.001), with significant variance in change (*p* = 0.012). Baseline performance was negatively associated with older age (β = −0.531, *p* < 0.001) and with changes in performance (*r* = −0.705, *p* < 0.001). Observations showed that a greater proportion of individuals improved (*n* = 174) rather than declined (*n* = 34). To investigate whether the improvement in working memory was driven by age, we conducted follow-up analyses after splitting the sample into two age groups based on the mean age of the sample (Mean = 50 years). The analyses revealed that the improvement was significant in younger (*n* = 99, Mean age 34; Mean change = 0.793, *p* = 0.021) but not among older adults (*n* = 109, Mean age = 64; Mean change = 0.478, *p* = 0.191).

### Influence of Striatal Iron on Working Memory and the Effects of Catechol-O-Methyltransferase

A model including striatal iron content and working memory exhibited an excellent fit, establishing strict invariance for working memory over time [χ^2^ (75, *n* = 208) = 65.26, *p* = 0.78, CFI = 1, RMSEA = 0.00 90% CI: 0.00–0.028]. Working memory performance improved over time (Mean = 6.02, *p* < 0.001), with significant variance in change (*p* = 0.017). Similarly, iron accumulated over time [Mean increase (susceptibility in ppm) = 0.019, *p* < 0.001], with significant variance in change (*p* < 0.001). Contrary to our hypotheses, baseline striatal iron did not predict working memory change (β = 0.009, *p* = 0.95) and greater iron accumulation was not significantly associated with working memory change (*r* = −0.08, *p* = 0.56). Figure 2A illustrates the results from the model.

There were no effects of *COMT* on baseline working memory or working memory change when contrasting Met homozygotes and heterozygotes (baseline working memory: β = 0.414, *p* = 0.08; working memory change: β = −0.473, *p* = 0.28) or Met and Val homozygotes (baseline working memory: β = −0.184, *p* = 0.42; working memory change: β = 0.035, *p* = 0.96). Likewise, there was no age x *COMT* interaction on working memory change contrasting Met homozygotes and heterozygotes (β = 0.632, *p* = 0.16), or Met and Val homozygotes (β = −0.114, *p* = 0.79).

Regarding the effects of *COMT* on baseline iron and iron accumulation, there were neither main effects on baseline iron (β = −0.269, *p* = 0.164) nor iron accumulation (β = 0.063, *p* = 0.81) and there were no age x *COMT* interactions (ps > 0.1) when contrasting Met homozygotes and heterozygotes. Similarly, there were no main effects of *COMT* on neither baseline iron nor iron accumulation when contrasting Met and Val homozygotes (baseline iron: β = −0.307, *p* = 0.108; iron accumulation: β = −0.301, *p* = 0.251). Whereas there was no age × *COMT* interaction on baseline iron (*p* = 0.183), there was a significant interaction on iron accumulation (β = 0.662, *p* = 0.012). To trace the source of the interaction, we conducted follow-up analyses after stratifying according to the two age groups. We contrasted Val and Met homozygotes first against each other, and then each homozygotes group against the heterozygotes group. The analysis revealed that in older age, Val homozygotes accumulated significantly more iron than Met homozygotes (β = 0.389, *p* < 0.001) and heterozygotes (β = 0.295, *p* = 0.006). By contrast, Met homozygotes did not differ from heterozygotes in older adults (β = 0.028, *p* = 0.80). Among younger adults, the difference between homozygotes with respect to iron accumulation did not significantly differ (β = 0.143, *p* = 0.259). As in old age, heterozygotes did neither differ from Met (β = 0.003, *p* = 0.98) nor Val homozygotes (β = 0.130, *p* = 0.29).

### Influence of Dorsolateral Prefrontal Cortex Iron on Working Memory and the Effects of Catechol-O-Methyltransferase

A model including working memory and DLPFC iron content exhibited an excellent fit, again establishing strict invariance for working memory over time [χ^2^ (75, *n* = 208) = 80.48, *p* = 0.31, CFI = 0.99, RMSEA = 0.019 90% CI: 0.00–0.045]. Baseline iron did not predict working memory change (β = 0.007, *p* = 0.96). As in the model with striatum, the mean changes in working memory performance were improved over time (Mean = 6.2, *p* < 0.001), with significant variance in change (*p* = 0.016). Similarly, iron accumulated over time (Mean increase of susceptibility in ppm = 0.014, *p* < 0.001), with significant variance in change (*p* < 0.001). As hypothesised, greater iron accumulation was associated with more deleterious changes in working memory (*r* = −0.312, *p* = 0.035). Figure 2B illustrates the results from the model.

Consistent with the striatal model, there were no effects of *COMT* on baseline working memory or working memory change when contrasting Met homozygotes and heterozygotes (baseline working memory β = 0.413, *p* = 0.79; working memory change β = −0.176, *p* = 0.65), or Met and Val homozygotes (baseline working memory β = −0.186, *p* = 0.42; working memory change β = 0.035, *p* = 0.93). Likewise, there was no age × *COMT* interaction on working memory change contrasting Met homozygotes and heterozygotes (β = 0.256, *p* = 0.51), or Met and Val homozygotes (β = 0.065, *p* = 0.86).

Regarding the effects of *COMT* on baseline iron and iron accumulation, there were no main effects of *COMT* when contrasting Met homozygotes and heterozygotes, but the effect on baseline iron was at trend level (baseline iron: β = −0.395, *p* = 0.075; iron accumulation: β = 0.391, *p* = 0.14). In comparison, when contrasting Met and Val homozygotes, *COMT* effect on baseline iron was not significant (β = −0.198, *p* = 0.37), whereas the effect on iron accumulation was marginally significant (β = −0.496, *p* = 0.056). There were no significant age × *COMT* interactions on baseline iron (Met homozygotes vs. Heterozygotes: β = 0.340, *p* = 0.127; Met homozygotes vs. Val homozygotes: β = 0.149, *p* = 0.50). However, contrasting Met and Val homozygotes revealed an age × *COMT* interaction effect on iron accumulation (β = 0.700, *p* = 0.007), and Met homozygotes and heterozygotes reached a marginally significant effect (β = −0.499, *p* = 0.059). Follow-up analyses revealed a significantly higher iron accumulation over time for Val compared to Met homozygotes in older (β = 0.217, *p* = 0.041), but not in younger adults (β = 0.034, *p* = 0.782). The comparison between Met homozygotes and heterozygotes did not reveal any difference with regard to iron accumulation in neither younger (β = 0.032, *p* = 0.798) nor older adults (β = −0.018, *p* = 0.867). However, Val homozygotes did differ from heterozygotes in older (β = 0.208, p = 0.05) but not in younger adults (β = −0.003, *p* = 0.98).

### Association Between Iron Accumulation in Dorsolateral Prefrontal Cortex and Working Memory Change as a Function of Age and Catechol-O-Methyltransferase

One of our main hypotheses was that older age may exacerbate the effect of lower levels of dopamine on brain iron accumulation and working memory. Based on the interaction between age and *COMT* on iron accumulation observed in DLPFC, we tested whether high levels of iron may be particularly detrimental in older Val carriers, reflected in a stronger correlation between iron accumulation and changes in working memory performance.

To investigate whether the change–change association would vary as a function of *COMT* and age, we extracted factor scores of the best fitting model involving DLPFC and working memory using regression imputation. Given the small sample sizes within groups, we conducted bootstrapping analyses to confirm the stability of associations and to avoid interpretation of spurious findings. We based the bootstrapping analyses on 5000 samples and reported bias-corrected 95% confidence intervals (CIs) of parameter estimates for the correlation coefficients. The effects of *COMT* were considered reliable if 95% CIs for the correlation coefficients did not include zeros. Independent of age, bivariate correlations revealed greater iron accumulation related to more deleterious change in working memory in both Val homozygotes (*r* = −0.352, *p* = 0.010, *n* = 53; BS 95% CI: −0.525/−0.165) and Met/Val heterozygotes (*r* = −0.421, *p* < 0.001, *n* = 92; BS 95% CI: −0.563/−0.263), but not in Met homozygotes (*r* = −0.02, *p* = 0.89, *n* = 54; BS 95% CI: −0.236/0.310).

**FIGURE 4:**
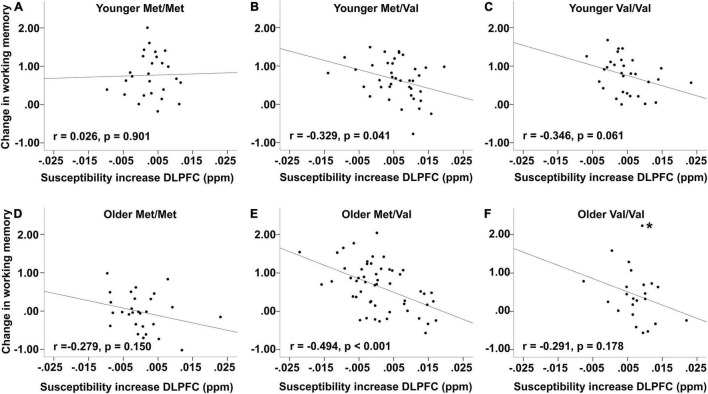
Association between changes in working memory and iron accumulation [susceptibility increase in parts per million (ppm)]. Top row: Younger **(A)** Met/Met, **(B)** Met/Val, and **(C)** Val/Val carriers. Bottom row: Older **(D)** Met/Met, **(E)** Met/Val, and **(F)** Val/Val carriers. Plots are based on imputed factor scores. * indicates marginal multivariate outlier whose exclusion led to a marginally significant association (see text).

Stratifying by age group, there were no significant correlations in neither younger nor older Met homozygotes (younger adults: *r* = 0.026, *p* = 0.901, *n* = 26, BS 95% CI: −0.437/0.462; older adults: *r* = −0.279, *p* = 0.15, *n* = 28, BS 95% CI: −0.701/0.173; Figures 4A,D). In contrast, both younger and older Met/Val heterozygotes showed significant negative association (younger adults: *r* = −0.329, *p* = 0.041, *n* = 39, BS 95% CI: −0.563/−0.074; older adults: *r* = −0.494, *p* < 0.001, *n* = 53, BS 95% CI: −0.701/−0.352; Figures 4B,E). However, Val homozygotes showed tendencies but did not reach conventional significance (younger adults: *r* = −0.364, *p* = 0.061, *n* = 30, BS 95% CI: −0.511/−0.182; older adults: *r* = −0.291, *p* = 0.178, *n* = 23, BS 95% CI: −0.557/0.016; Figures 4C,F). Note that one marginal multivariate outlier was discovered among older Val homozygotes. Excluding this marginal outlier would result in a marginally significant association (*r* = −0.406, *p* = 0.061, *n* = 22, BS 95% CI: −0.683/0.088).

Note, however, that none of the associations between younger and older adults across *COMT* status were significantly different from each other (all *p*-values > 0.1).

## Discussion

We investigated whether genetic predispositions associated with low levels of dopamine would be associated with higher brain iron accumulation and working memory change in an adult lifespan sample. Our main finding was that iron accumulated over time in both striatum and DLPFC, and this accumulation was amplified in older *COMT* Val homozygotes who presumably have the lowest endogenous dopamine levels. We also found that more iron accumulation in DLPFC was associated with more deleterious change in working memory. Specifically, this link was driven by Val carriers.

### Dorsolateral Prefrontal Cortex Iron Accumulation and Working Memory Change

In addition to our novel finding of a significant accumulation of iron in DLPFC over time, we replicated the finding of accumulation of iron in striatum in line with two other longitudinal studies (Daugherty et al., 2015; Daugherty and Raz, 2016). In both striatum and DLPFC, greater baseline iron content was associated with older age and less iron accumulation, suggesting a deceleration of iron accumulation across the lifespan. In line with cross-sectional data, we observed higher iron values in striatum than in DLPFC (Hallgren and Sourander, 1958; Acosta-Cabronero et al., 2016; Zhao et al., 2019). The exact factors behind the distribution of iron concentration remain elusive, but it has been speculated that regional difference in iron load may reflect regional differences in iron demand (c.f. Zhao et al., 2019).

A major finding of the present study is the association between higher iron accumulation in DLPFC and more detrimental working-memory change. The link between iron accumulation and changes in cognition is poorly understood as longitudinal studies are sparse. Our finding of greater iron accumulation in DLPFC being related to a more deleterious impact on working memory change across approximately 3 years extends the previous evidence of the detrimental effect of brain iron overload on cognition mostly based on cross-sectional studies (Rodrigue et al., 2013; Ghadery et al., 2015). In contrast with previous cross-sectional and longitudinal findings, we did not observe a relationship between iron in striatum and working memory (Daugherty et al., 2015; Blasco et al., 2017; Salami et al., 2021). Both DLPFC and striatum are crucial for working memory performance (for review, see Eriksson et al., 2015). Surprisingly, only iron accumulation in DLPFC was associated with changes in working memory. In contrast to Daugherty et al. (2015), who showed baseline iron predicting working memory change, no such pattern was observed in our study for neither striatum nor DLPFC. Nevertheless, the differences between the present and previous findings are not mutually exclusive, they may instead reflect the same chain of events but at different stages. The pattern of baseline iron predicting change in working memory could reflect that a specific threshold of iron load may be needed for detrimental effects on cognition to occur (c.f. Kalpouzos et al., 2017), which may manifest only with advancing age. In our findings, that threshold may have been reached between the two time points, manifesting in a change-change association. The discrepancy between the two studies may also be due to substantial other differences, such as the difference in sample size (*n* = 125 vs. *n* = 208 at baseline; *n* = 78 vs. *n* = 135 at follow-up), method of assessing iron (R2* vs. QSM), and measurement of working memory (two verbal and two non-verbal vs. three non-verbal tasks).

### Greater Iron Accumulation in Catechol-O-Methyltransferase Val Homozygotes

A key finding in the present study is that older *COMT* Val homozygotes accumulated more iron in DLPFC and striatum compared to Met carriers, suggesting that lower levels of dopamine may exacerbate the accumulation of iron in these regions. The reason we see a difference in iron accumulation between *COMT* genotypes in older adults might be due to less available dopamine, as older adults have been observed to have lower pre- and postsynaptic markers of dopamine (dopamine transporter and receptor availability, respectively; Bäckman et al., 2006, 2010 for review). Based on the *in vitro* literature reporting the important role of dopamine in cellular iron homoeostasis (Dichtl et al., 2018; Liu et al., 2021), the changes in iron homoeostasis may be exacerbated in Val carriers with age-related decrease of dopamine. The COMT enzyme is important for controlling degradation of endogenous dopamine and accounts for over 60% of total dopamine turnover in DLPFC and 15% in striatum (Karoum et al., 1994). As the uptake of free iron (Dichtl et al., 2018) and suppression of neuroinflammation (Shao et al., 2013; Yan et al., 2015; Zhu et al., 2018) decreases with less dopamine available, the transport and release of bound iron might increase in an attempt to return to homoeostasis, leading to more oxidative stress and creating a deleterious loop (Mills et al., 2010; Ward et al., 2014). As young adults have more intact dopaminergic systems, this cycle of increased oxidative stress may be manageable. Nevertheless, our results of greater iron accumulation among older Val homozygotes is in line with the magnification hypothesis in old age previously reported on white-matter structure and brain activity (Sambataro et al., 2009; Papenberg et al., 2015).

### Iron Accumulation and Working-Memory Changes as a Function of Age and Catechol-O-Methyltransferase

Working memory performance improved over time in our sample. However, these improvements were only significant among younger adults and not among the older adults. It is possible that the improvement observed among younger adults was due to practice effects, which may be present for several years (Rönnlund et al., 2005). It is likely that there are also practice effects among older adults, which might be overshadowed by stability or less improvement in working memory over time, thus resulting in lack of change in performance.

The lack of an effect of *COMT* on cognition may not be surprising, as some studies have reported an effect (De Frias et al., 2004, 2005), whereas others have not (Bolton et al., 2010; Blanchard et al., 2011; Papenberg et al., 2014), suggesting that *COMT* has limited effects on cognition or not big enough sample to see any effect (see Barnett et al., 2007, 2008 for meta-analytic evidence). One explanation might be that the negative effects of having lower levels of dopamine may be only detrimental to cognition in light of high postsynaptic receptor availability (Lövdén et al., 2018; Papenberg et al., 2020). An inverted u-shape function of dopamine signalling and cognition suggests that excessively low as well as excessively high dopamine is related to poorer cognitive performance (Cools and D’Esposito, 2011). Optimal balance between endogenous dopamine and receptor availability would reflect an equilibrium, which would also be optimal for performance when both dopamine and receptor availability are low. More specifically, Val homozygotes with matched dopamine availability and D2DR status (i.e., low dopamine transmitter and low D2 receptor availability) showed higher levels of memory performance compared to Val homozygotes with high D2DR status or even Met homozygotes with low D2DR status (i.e., insufficient or excessive dopamine transmitter availability in relation to receptor availability; Papenberg et al., 2020). As such, positive and negative effects of being Val carrier on cognition may be cancelled out as they depend on the number of receptors. *COMT* Val might drive iron accumulation, but the negative effect on cognition might be attenuated by the receptor differences and would be more visible with advancing age as receptor availability further decrease (Bäckman et al., 2006, 2010) and the detrimental effect of iron on cognition is exacerbated (Kalpouzos et al., 2017; Salami et al., 2021).

Although we did not observe a direct effect of *COMT* on cognition, the observation that those who accumulated more iron in DLPFC also showed downtrending changes in working memory among Val carriers can be interpreted as an emerging effect of *COMT*. In Val carriers, the negative association between iron accumulation and working-memory change might reflect impending working-memory decline, similarly to cognitive decline among Val carriers reported in previous studies (De Frias et al., 2004, 2005). That is, Val carriers are presumably characterised by the lowest levels of dopamine. This, in turn, might lead to more iron accumulation and become more deleterious for working memory, as performance would become increasingly impaired with time.

### Strengths and Limitations

Our results should be interpreted within the strengths and limitations of our study. First, our study utilised longitudinal data capturing the adult lifespan, which allowed us to address novel questions in an underinvestigated field of research pertaining to dopamine and brain iron. A common problem with longitudinal studies is attrition over time. However, using full information maximum likelihood, in which all available data is used to estimate missing data, the results of the population estimates should be less biassed and more precise (Schafer and Graham, 2002).

Second, using structural equation modelling allows for testing of complex patterns of relationships between multiple variables and groups which would otherwise require several separate analyses. SEM allows for the estimate of latent variables from observed variables and takes into account measurement and reliability errors in the data, leading to less biassed estimates.

Third, QSM is a recently developed technique for quantitative estimate of tissue magnetic susceptibility and has demonstrated good reproducibility (Wang and Liu, 2015) and validity (Langkammer et al., 2012; Sun et al., 2015) as an approximation of *in vivo* iron. The strength of QSM is that it removes non-local background susceptibility effects and allows for the separation of magnetic properties. As such, the diamagnetic properties of myelin or calcifications can be distinguished from the paramagnetic properties of iron by QSM, in comparison to other methods such as R2*. While post-mortem studies have validated QSM as a proxy for iron (Langkammer et al., 2012), with a correlation of *r* = 0.84 between QSM and chemically determined iron concentration in grey matter, it has to be acknowledged that other minerals [e.g., copper, manganese, and calcium, albeit contribution may be small (Schenck and Zimmerman, 2004), presence of microbleeds, and myelin may contribute to the averaged QSM signal within a region of interest given their diamagnetic or paramagnetic properties (Haacke et al., 2005; Sedlacik et al., 2007; Schweser et al., 2011; Lee et al., 2012; Stüber et al., 2014; Miletić et al., 2022)]. Presence of diamagnetic elements causes R2* to overestimate iron content and QSM to underestimate it (Haacke et al., 2005). Although both QSM and R2* were available, for the present study we favoured QSM over R2*, as Wenger et al. (2021) recently showed poor reliability of R2* in regions outside basal ganglia (see section “MRI Acquisition and Preprocessing” Methods for further detail).

The small size of our sample could have limited the power to attain conventional statistical significance in subgroup analyses (e.g., *n* = 23 older Val/Val), and further restricted the possibility to run multigroup analyses as a function of *COMT*. A larger sample size would have enabled a comparison of the groups in latent space and allowed to examine possible cohort effects on iron accumulation and changes in working memory using more equally distributed age groups across the lifespan.

## Conclusion

Our study contributes to a mechanistic understanding of how dopamine may modulate iron accumulation across the human lifespan. Individual differences in genetic predisposition related to lower dopamine levels appeared to increase the accumulation of brain iron in older adults, which in turn, impacted working memory change in a deleterious fashion. This link, which was more pronounced among individuals with low dopamine levels (i.e., *COMT* any Val carriers), may indicate an early marker for oncoming cognitive decline among Val carriers, underlining the importance of further investigation. Future studies are necessary to better understand the role of the dopaminergic system in brain and cognitive ageing, for example, by including biomarkers such as dopamine D1 and D2 receptor availability and receptor-related genes. Moreover, alternative iron-rich regions involved in dopaminergic pathways and cognition, such as substantia nigra, subthalamic nucleus, and globus pallidus would be interesting to investigate to further understand the dopamine-iron link.

## Data Availability Statement

The data that support the findings of this study are available from the corresponding author upon reasonable request.

## Ethics Statement

The studies involving human participants were reviewed and approved by the Regional Ethical Review Board in Stockholm. The participants provided their written informed consent to participate in this study.

## Author Contributions

GP and GK designed and performed the research. JG and GK collected the data. JG, GP, FF, EL, and GK analysed and interpreted the data. JG, FF, and GP wrote the manuscript which was edited by all authors.

## Conflict of Interest

The authors declare that the research was conducted in the absence of any commercial or financial relationships that could be construed as a potential conflict of interest.

## Publisher’s Note

All claims expressed in this article are solely those of the authors and do not necessarily represent those of their affiliated organizations, or those of the publisher, the editors and the reviewers. Any product that may be evaluated in this article, or claim that may be made by its manufacturer, is not guaranteed or endorsed by the publisher.

## Footnotes

1
http://weill.cornell.edu/mri/pages/qsm.html

2
http://fsl.fmrib.ox.ac.uk

3
http://surfer.nmr.mgh.harvard.edu/
